# Quantitative Trait Locus Analysis and Identification of Candidate Genes for Micronaire in an Interspecific Backcross Inbred Line Population of *Gossypium hirsutum* × *Gossypium barbadense*

**DOI:** 10.3389/fpls.2021.763016

**Published:** 2021-10-27

**Authors:** Wenfeng Pei, Jikun Song, Wenkui Wang, Jianjiang Ma, Bing Jia, Luyao Wu, Man Wu, Quanjia Chen, Qin Qin, Haiyong Zhu, Chengcheng Hu, Hai Lei, Xuefei Gao, Haijun Hu, Yu Zhang, Jinfa Zhang, Jiwen Yu, Yanying Qu

**Affiliations:** ^1^Engineering Research Centre of Cotton of Ministry of Education, College of Agriculture, Xinjiang Agricultural University, Urumqi, China; ^2^State Key Laboratory of Cotton Biology, Key Laboratory of Cotton Genetic Improvement, Ministry of Agriculture, Institute of Cotton Research, Chinese Academy of Agricultural Sciences, Anyang, China; ^3^Zhengzhou Research Base, State Key Laboratory of Cotton Biology, Zhengzhou University, Zhengzhou, China; ^4^Western Agriculture Research Centre, Chinese Academy of Agricultural Sciences, Changji, China; ^5^Seed Management Station, Department of Agriculture and Rural Affairs of Xinjiang, Urumqi, China; ^6^Join Hope Seed Co., Ltd., Changji, China; ^7^Department of Plant and Environmental Sciences, New Mexico State University, Las Cruces, NM, United States

**Keywords:** *Gossypium hirsutum*, *Gossypium barbadense*, backcross inbred lines, micronaire, quantitative trait locus

## Abstract

Cotton is the most important fiber crop and provides indispensable natural fibers for the textile industry. Micronaire (MIC) is determined by fiber fineness and maturity and is an important component of fiber quality. *Gossypium barbadense* L. possesses long, strong and fine fibers, while upland cotton (*Gossypium hirsutum* L.) is high yielding with high MIC and widely cultivated worldwide. To identify quantitative trait loci (QTLs) and candidate genes for MIC in *G. barbadense*, a population of 250 backcross inbred lines (BILs), developed from an interspecific cross of upland cotton CRI36 × Egyptian cotton (*G. barbadense*) Hai7124, was evaluated in 9 replicated field tests. Based on a high-density genetic map with 7709 genotyping-by-sequencing (GBS)-based single-nucleotide polymorphism (SNP) markers, 25 MIC QTLs were identified, including 12 previously described QTLs and 13 new QTLs. Importantly, two stable MIC QTLs (*qMIC-D03-2* on D03 and *qMIC-D08-1* on D08) were identified. Of a total of 338 genes identified within the two QTL regions, eight candidate genes with differential expression between TM-1 and Hai7124 were identified. Our research provides valuable information for improving MIC in cotton breeding.

## Introduction

Cotton (*Gossypium* spp.) is an important cash crop species worldwide, providing an essential natural resource for the textile industry. Due to its high yield and wide adaptation, upland cotton (*Gossypium hirsutum* L.) accounts for more than 95% of global cotton production (Lacape et al., 2003; Li X. M. et al., 2016). However, extra-long staple, Pima, Egyptian, or Sea Island cotton (*Gossypium barbadense* L.) have excellent fiber quality with long, strong and fine fibers, but their low yield and requirements for warm and dry weather conditions limit their cultivation area (Zhang et al., 2014; Said J. I. et al., 2015). In recent years, the goal of cotton breeding in China has shifted to improving fiber quality (including fiber length, strength, and micronaire (MIC)], in addition to high yield (Fang et al., 2017). To date, there have been an increasing number of studies on improving cotton fiber quality traits through interspecific hybridization, especially *G. hirsutum* × *G. barbadense* (Zhang et al., 2014; Wang et al., 2020).

Cotton fibers are the longest and fastest growing cells of cotton plants. Each cotton fiber consists of a single cell that grows on the surface of the ovule. The fiber development process includes four main stages: fiber initiation, elongation, secondary wall thickening and maturation (Pang et al., 2010). Cotton fiber quality is a quantitative trait affected by multiple genes (genotype), environmental factors and genotype × environment interactions during fiber development. MIC is mainly determined by the formation characteristics of fiber secondary walls (Wu et al., 2020). Its value is determined by measuring the airflow resistance of a certain weight of cotton fiber plug (i.e., μg per inch of single fiber). Textile processing companies and scientific research organizations have adopted MIC as a key parameter of fiber maturity and fineness (Bradow and Davidonis, 2000). MIC is a comprehensive index of fiber fineness and maturity for fiber quality and plays an important role in the fiber spinning process. Because immature fibers have thin cell walls and therefore low MIC (below 3.4), they tend to become weaker and easily break during the spinning process, making low grade yarns. However, mature fibers have thick cell walls and produce a thick yarn. So mature cotton fibers are preferred in spinning (Kim et al., 2013). For mature fibers, MIC reflects the fineness of the fibers in that the higher the MIC, the coarser the mature fibers. Mature fibers with MIC readings between 3.70 and 4.20 are considered premium. However, micronaire readings of 3.4- and -under or 5.0- and -higher will receive discount in pricing. Therefore, it is of great theoretical and applied value to analyze and identify candidate genes regulating MIC at the quantitative trait locus (QTL) level for fiber quality molecular breeding and elucidate the genetic mechanism underlying cotton fiber development.

Quantitative trait locus mapping uses molecular marker technology as a tool based on genetic linkage maps and uses the linkage between linked molecular markers and QTLs to determine the position of candidate genes that control quantitative traits throughout the genome. At present, two commonly used methods include composite interval mapping (CIM) and inclusive composite interval mapping (ICIM) (Meng et al., 2015). Software used for these two methods include WinQTLCart 2.5 for CIM and QTL IciMapping 4.2 for ICIM. Most researchers have used these two software programs separately to carry out QTL mapping research on important cotton traits. For example, CIM was used by Li C. et al. (2016), Zhang et al. (2016) and Liu et al. (2018), and ICIM was used by Liu et al. (2017, 2019) and Ma et al. (2017). However, these two mapping methods can be simultaneously used for locating QTLs to perform a more accurate and comprehensive genetic analysis of traits.

Using one of the two methods, studies have also reported QTLs for MIC. Ali et al. (2018) identified 22 MIC-related QTLs in a RIL population of 180 lines in upland cotton, among which 13 QTLs were detected in two or more environments. Wang B. H. et al. (2017) detected 27 MIC-related QTLs using BC_3_F_2_, BC_3_F_2__:__3_, and BC_3_F_2__:__4_ populations of an interspecific *G. hirsutum* × *Gossypium mustelinum* cross, among which 11 QTLs were located near the same marker in different populations or near linked markers in the same population. In addition, Fan et al. (2018) identified four MIC-related QTLs using a population of 143 RILs of an intra-*G. barbadense* cross. With the rapid development of genome sequencing technology, genome-wide association study (GWAS) has been successfully applied in the genetic analysis of fiber quality traits, including fiber MIC. Using genome resequencing, Wang M. J. et al. (2017) identified 3 significant single-nucleotide polymorphisms (SNPs) for fiber MIC in a group of 362 diverse upland cotton accessions, and Ma et al. (2018) identified 533 significant SNPs for fiber MIC in a panel of 419 upland cotton accessions. In addition, Huang et al. (2017) used the cotton Illumina 63K SNP array to genotype a collection of 503 upland cotton lines and identified 3 stable QTLs associated with MIC. Through a meta-analysis of numerous QTL reports, Said J. et al. (2015) compiled a total of 395 QTLs related to MIC in a QTL database for cotton.^1^ Xu et al. (2020) recently performed a meta-analysis and identified a total of 15 meta-QTLs for MIC. These studies provide references for locations of QTLs for MIC.

Although *G. barbadense* has much longer, stronger and finer fibers than *G. hirsutum*, whether there exist major QTLs for MIC when crossing with *G. hirsutum* is currently not well understood. In this study, we used a population of 250 backcross inbred lines (BILs) from a *G. barbadense* × *G. hirsutum* cross (Ma J. et al., 2019) and identify QTLs for MIC based on a high-quality genetic map using two QTL mapping methods. The identified QTLs were then subjected to an integrated analysis to identify BILs with low MIC (i.e., fine fibers) and candidate genes for MIC. The results will lay the foundation for subsequent fine mapping of MIC-related genes and molecular marker-assisted selection (MAS) to improve MIC in upland cotton.

## Materials and Methods

### Plant Materials

An interspecific BIL population comprising 250 BC_1_F_7_ lines was developed from a cross between Egyptian cotton (*G. barbadense*) Hai7124 and Chinese *G. hirsutum* CRI36. The parents and 250 BC_1_F_7_ lines were planted in accordance with a randomized complete block design in nine environments at five locations, including the South Farm (nc) and the East Farm (dc) at the Institute of Cotton Research, Chinese Academy of Agricultural Sciences (CRI-CAAS), Anyang, Henan, China (Aync, 2015, 2016, 2017, and Aydc, 2017); Weixian, Hebei (Hbwx, 2016); Sanya, Hainan (Hnsy, 2016); and Alar, south Xinjiang (Xjal, 2016, 2017); and Shihezi, Northern Xinjiang (Xjsh, 2017). Crop management practices followed local recommendations for cotton production. The use of two major cotton production regions (the Yellow River Valley and Northwestern Inland Valley) allowed the detection of consistent QTLs for MIC between the two regions. The specific length amplified fragment sequencing (SLAF-seq) strategy was followed for genotyping the BILs using a *G. hirsutum* reference genome with updates (Zhang et al., 2015; Hu et al., 2019). The details of a genetic linkage map consisting of 7709 markers were described previously by Ma J. et al. (2019).

### Phenotypic Measurements and Analysis

Twenty normally mature (opened) bolls from the first and second nodes of middle fruiting branches were sampled in September each year. All seedcotton samples were ginned by a roller gin in the South Farm at CRI-CAAS, Anyang, Henan. Fiber samples were then tested by an HVI 1000 instrument at the Cotton Quality Inspection and Supervision Center of the Ministry of Agriculture, CRI-CAAS, Anyang, Henan. The R software package lme4 was used to estimate the best linear unbiased predictions (BLUPs) and broad-sense heritability (*H*^2^) for MIC across the nine environments (Bates et al., 2014). The R software was also used for other statistical analyses including analysis of variance (ANOVA) and principal component analysis (PCA) of MIC for the BIL population across different environments.

### Quantitative Trait Locus Analysis

Micronaire values in each of the nine testing environments and their BLUPs across the tests were used for QTL analysis using the ICIM of ADDitive QTL (ICIM-ADD) method in QTL IciMapping 4.2 (Meng et al., 2015) and the CIM method in WinQTLCart 2.5 (Wang et al., 2007). The parameters were set to a mapping step of 1 cM, a *p* value of 0.05 for type I error, and a PIN of 0.01, and 1000 permutations were taken to calculate the logarithm of odds (LODs) threshold. QTLs at the same location in two or more environments with a LOD threshold of >2.5 were considered significant QTLs (Shang et al., 2015). The QTL confidence interval (95%) was set as a mapping distance interval corresponding to a decrease of 1 LOD on either side of the peak (Yu et al., 2012a; Liu et al., 2019). MIC QTLs detected in three or more environments were considered stable QTLs when their confidence intervals overlapped (Yu et al., 2012b; Ma J. et al., 2019). A set of consensus QTLs for MIC was inferred by integrating the information of QTLs detected via the two methods. QTLs were named according to the method of Sun et al. (2013), with a prefix of *W*, *I*, and *C* for a QTL identified by CIM, ICIM and both methods, respectively. MapChart 2.2 was used to visualize the genetic map and QTL bars.

### Common Quantitative Trait Loci for Micronaire in the Backcross Inbred Line Population and Previous Reported Studies

To identify new QTLs in this study, QTLs from our results were compared with previously reported QTLs. Previous MIC QTLs were retrieved from CottonGen (Yu et al., 2014) and Cotton QTLdb Release 2.3 (January 24, 2018, see text footnote 1) (Said J. et al., 2015) and from recent reports by Majeed et al. (2019) and Xu et al. (2020). In addition, MIC QTL data from previous GWAS reports were also obtained. A co-localizing marker or a neighboring marker for a MIC QTL was identified, and then the marker location on the TM-1 genome was determined (Zhang et al., 2015; Hu et al., 2019). The physical intervals of all QTLs were queried via BLAST against the TM-1 genome, and QTLs were co-localized together with the previously identified MIC-related QTLs.

### Gene Ontology Enrichment and Candidate Gene Identification

After the physical intervals of stable QTLs were queried via BLAST against the TM-1 genome (Hu et al., 2019), potential candidate genes were determined on the basis of the physical interval for a QTL. The homologous genes of candidate genes from *Arabidopsis* and the annotations of gene functions were determined from the TM-1 genome. The general pattern of expression of the candidate genes and their SNPs including insertion/deletion (InDel) of TM-1 and Hai7124 were also obtained from Hu et al. (2019) and then analyzed by SnpEff to predict variant impact (Cingolani, 2012). Gene Ontology (GO) enrichment of candidate genes was performed using the micStudio tools.^2^ Homologous genes of candidate genes from *Arabidopsis* were used to determine enriched ontology clusters by Metascape (Zhou et al., 2019). Candidate genes were further used to predict the micro-RNA (miRNA) target genes by psRNATarget^3^ (Dai et al., 2018), and the miRNA expression data of fibers at 14 days post-anthesis (DPA) were obtained from the Cotton Omics Database.^4^

## Results

### Micronaire Variation of Parents and the Backcross Inbred Line Population

Across the nine environments, MIC of the BILs ranged from 2.10 to 6.17, with an average of 4.14, and the mean MIC of the Upland CIR36 and Egyptian Hai7124 parents were 3.59 and 4.06, respectively (Table 1). The MIC of CIR36 was significantly (*P* < 0.01) greater than that of Hai7124. The values of skewness and kurtosis in each environment showed that MIC followed a normal distribution in the BIL population (Supplementary Figure 1 and Table 1). Furthermore, there was a transgressive segregation of MIC within the BIL population compared with the Hai7124 and CRI36 parents. The ANOVA detected significant variations in MIC (*P* < 0.01) due to environment and genotype in the BIL population (Supplementary Table 1). However, the *H*^2^ estimate for MIC (i.e., the percentage of the total phenotypic variance accounted for by the genotypic variance) was 93.44%, suggesting that MIC was highly heritable in this BIL population (Supplementary Table 1).

**TABLE 1:** Performance of backcross inbred lines (BILs) of Hai7124 × CRI36 hybrids and their parents.

Trait	Test	Parent		BILs			SD	Skewness	Kurtosis	CV (%)
		Hai7124	CRI36	Min	Max	Mean				
MIC	Aync (2015)	3.40	3.80*	2.10	5.70	3.84	0.75	0.17	–0.34	19.45
	Aync (2016)	3.67	4.33*	2.89	5.51	4.45	0.60	–0.33	–0.39	13.42
	Hnsy (2016)	3.30	4.50*	2.20	5.90	3.66	0.66	0.57	0.17	17.92
	Xjal (2016)	nt	nt	2.55	6.07	4.04	0.61	0.24	0.38	15.21
	Aydc (2017)	4.22	4.37*	2.57	6.14	4.46	0.64	–0.10	–0.35	14.24
	Aync (2017)	3.91	4.17*	2.43	6.17	4.52	0.66	–0.12	–0.19	14.65
	Hbwx (2017)	3.91	4.10*	2.37	5.87	4.49	0.57	–0.28	0.28	12.64
	Xjal (2017)	3.12	3.96*	2.35	5.59	3.85	0.62	0.22	–0.10	16.08
	Xjsh (2017)	3.16	3.27*	2.28	5.84	3.99	0.72	–0.03	–0.52	18.04

*Difference was significant at P < 0.05 when the two parents were compared. nt, not tested. Anyang, Henan (Aync, 2015, 2016, 2017, and Aydc, 2017); Weixian, Hebei (Hbwx, 2016); Sanya, Hainan (Hnsy, 2016); and Alar, Xinjiang (Xjal, 2016, 2017); and Shihezi, Xinjiang (Xjsh, 2017).

**FIGURE 1:**
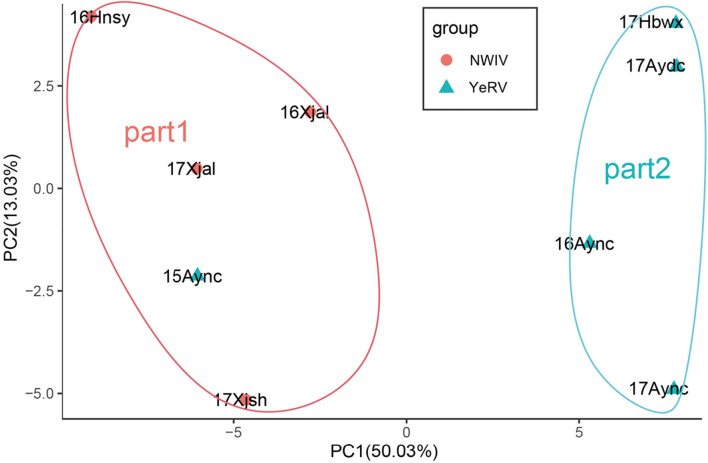
Principal component analysis (PCA) analysis of MIC from 250 BILs in nine environments. 16Aync, 17Aync, 17Aydc, and 17Hbwx environment were classed as region2 by PCA which represented to the Yellow River Valley (YeRV) cotton growing regions. 16Xjal, 17Xjal, and 17Xjsh environment with 15Aync and 16Hnsy were classed as region1 by PCA which nearly represented to the Northwest Inland Valley (NWIV) cotton growing regions.

Principal component analysis of the MIC value in this set of BILs showed that the nine environments could be classified into two regions: Region 1 (Northwestern Inland Valley) and Region 2 (Yellow River Valley) (Figure 1), mostly consistent with the official ecological classification of the cotton production regions in China, except for two tests-Anyang, Henan, 2015 (15Aync) and Sanya, Hainan, 2016 (16Hnsy) which were grouped with Region 1. Therefore, the testing environments of the two regions were separately estimated using BLUPs as BLUP-region 1 and BLUP-region 2.

### Quantitative Trait Loci for Micronaire in the Backcross Inbred Lines via Composite Interval Mapping

**FIGURE 2:**
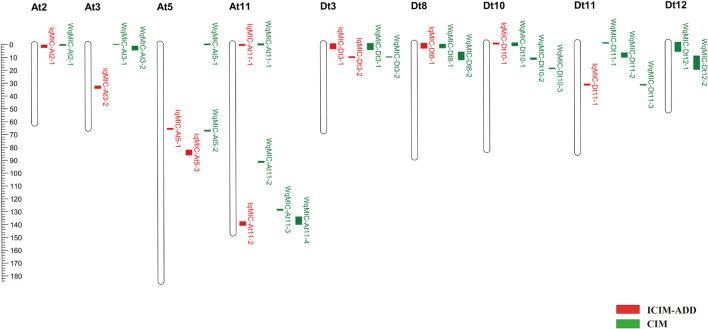
The chromosome-wise distribution of QTL for MIC by ICIM and CIM.

The nine testing environments and three BLUPs including BLUP across the nine environments, BLUP region 1 and BLUP region 2 were used for a total of 12 QTL analyses (or tests). In total, 21 QTLs (9 on the A subgenome and 12 on the D subgenome) for MIC were detected on 9 chromosomes by CIM (Figure 2 and Supplementary Table 2), and each QTL explained 5.08–16.56% of the phenotypic variation with LOD scores varying from 3.65 to 10.17. Six QTL alleles from Hai7124 (*WqMIC-At2-1*, *WqMIC-Dt8-1*, *WqMIC-Dt8-2*, *WqMIC-Dt11-3*, *WqMIC-Dt12-1*, and *WqMIC-Dt12-2*) had positive additive effects on MIC (i.e., increasing MIC), while other 15 QTL alleles from Hai7124 (*WqMIC-At3-1*, *WqMIC-At3-2*, *WqMIC-At5-1*, *WqMIC-At5-2*, *WqMIC-At11-1*, *WqMIC-At11-2*, *WqMIC-At11-3*, *WqMIC-At11-4*, *WqMIC-Dt3-1*, *WqMIC-Dt3-2*, *WqMIC-Dt10-1*, *WqMIC-Dt10-2*, *WqMIC-Dt10-3*, *WqMIC-Dt11-1*, and *WqMIC-Dt11-2*) had negative additive effects on MIC (i.e., decreasing MIC). Importantly, three QTLs (*WqMIC-Dt3-1*, *WqMIC-Dt3-2*, and *WqMIC-Dt8-1*) were consistently identified in at least three tests and were declared stable QTLs; and other 18 QTLs were detected in one or two tests.

### Quantitative Trait Loci for Micronaire in the Backcross Inbred Lines via Inclusive Composite Interval Mapping

In total, 12 QTLs for MIC were detected on 8 chromosomes across 12 tests by ICIM-ADD (Figure 2 and Supplementary Table 3), each of which explained 3.20–12.56% of the phenotypic variation with LOD scores between 2.62 and 10.32. Among the 12 QTLs, 7 and 5 QTLs were identified on the A subgenome and D subgenome, respectively. Six QTL alleles from Hai7124 (*IqMIC-At2-1*, *IqMIC-At3-2*, *IqMIC-At5-2*, *IqMIC-Dt8-1*, and *IqMIC-Dt11-1*) had positive additive effects on MIC, while other six QTL alleles from Hai7124 (*IqMIC-At3-1*, *IqMIC-At5-1*, *IqMIC-At11-1*, *IqMIC-At11-2*, *IqMIC-Dt3-1*, *IqMIC-Dt3-2*, and *IqMIC-Dt10-1*) had negative additive effects on MIC. Importantly, four QTLs (*IqMIC-At5-2*, *IqMIC-At11-1*, *IqMIC-Dt3-2*, and *IqMIC-Dt8-1*) were consistently identified in at least three tests and were declared stable QTLs; and other eight QTLs were detected in one or two tests.

Between 21 MIC QTLs detected by CIM and 12 MIC QTL detected by ICIM, eight QTLs (*CqMIC-At2-1*, *CqMIC-At11-1*, *CqMIC-At11-4*, *CqMIC-Dt3-1*, *CqMIC-Dt3-2*, *CqMIC-Dt8-1*, *CqMIC-Dt10-1*, and *CqMIC-Dt11-1*) were commonly identified by both QTL mapping methods, because they shared overlapping confidence intervals. The 2 stable QTLs- *CqMIC-Dt3-2* and *CqMIC-Dt8-1* mapped by both methods were simplified as *qMIC-Dt3-2* and *qMIC-Dt8-1*, respectively, in the following analysis. Both methods detected MIC QTLs on eight common chromosomes (At02, At03, At05, At11, Dt03, Dt08, Dt10, and Dt11), in addition to two QTLs on Dt12 detected by CIM. On these common chromosomes with QTLs detected by both methods, most of them (8/12) detected by ICIM were also detected by CIM, and a few of them (4/12) had different mapping positions than these detected by CIM. However, CIM detected more QTLs on these chromosomes. The results suggest that both methods can detect unique QTLs, but CIM may detect more QTLs than that ICIM. Overall, a total of 25 QTLs were detected by the combined use of the two QTL mapping methods (Table 2 and Figure 2). A total of 12 and 13 MIC QTLs were distributed on the At and Dt subgenomes, respectively. Interestingly, 13 QTLs were detected on two pairs of homeologous chromosomes (4 on At03 vs. 2 on Dt03, and 4 on At11 vs. 3 on Dt11). It appears that they were not distributed on homeologous chromosome regions.

**TABLE 2:** Summary of micronaire (MIC) QTLs identified in different environments by ICIM-ADD and CIM.

Meta-QTL name	QTL name^a^	LOD	PVE (%)	Add	Position (cM)	95% Confidence	No. tests	Reported previously
*CqMIC-At2-1*	*IqMIC-At2-1*	3.63	5.16	–0.16	61.5	60.5–62.5	2	
	*WqMIC-At2-1*	3.86	5.56	–0.18	61.7	60–63.4	2	
*CqMIC-At3-1*	*WqMIC-At3-1*	3.97	5.84	0.18	89.3	89.1–89.5	2	
*CqMIC-At3-2*	*WqMIC-At3-2*	4.70	6.12	0.15	91.9	90.6–93.8	1	
*CqMIC-At3-1*	*IqMIC-At3-1*	10.32	12.56	0.20	103.0	102.5–103.5	1	
*CqMIC-At3-2*	*IqMIC-At3-2*	4.10	4.66	–0.11	123.0	121.5–123.5	1	
*CqMIC-At5-1*	*WqMIC-At5-1*	4.33	7.02	0.24	0.0	0–0.8	1	Guo et al., 2007
*CqMIC-At5-2*	*IqMIC-At5-1*	5.09	8.89	0.22	66.0	65.5–66.5	2	Xiao et al., 2009; Tang et al., 2014; Huang et al., 2017; Latyr et al., 2018; Tan et al., 2018
*CqMIC-At5-3*	*WqMIC-At5-2*	9.22	16.04	0.29	67.9	67–68	1	Xiao et al., 2009; Tang et al., 2014; Huang et al., 2017; Latyr et al., 2018; Tan et al., 2018
*CqMIC-At5-4*	*IqMIC-At5-3*	3.10	4.22	–0.14	85.3	82.5–86.5	3	Xiao et al., 2009; Tang et al., 2014; Huang et al., 2017; Latyr et al., 2018; Tan et al., 2018
*CqMIC-At11-1*	*IqMIC-At11-1*	3.75	5.95	0.18	18.0	17.5–18.5	6	Guo et al., 2007
	*WqMIC-At11-1*	3.84	5.08	0.15	17.2	17.1–18.3	1	
*CqMIC-At11-2*	*WqMIC-At11-2*	4.18	6.43	0.21	109.1	108.5–109.6	1	Guo et al., 2007
*CqMIC-At11-3*	*WqMIC-At11-3*	3.95	5.75	0.19	146.8	145.8–146.9	1	Tang et al., 2014
*CqMIC-At11-4*	*WqMIC-At11-4*	4.44	8.27	0.28	155.4	151.9–157.9	1	Tang et al., 2014
	*IqMIC-At11-2*	4.31	8.71	0.29	156.0	155.5–158.5	1	
*CqMIC-Dt3-1*	*IqMIC-Dt3-1*	5.89	10.15	0.26	9.0	6.5–10.5	2	Rong et al., 2004; Jia et al., 2018; Latyr et al., 2018
	*WqMIC-Dt3-1*	6.90	10.74	0.24	11.1	6.3–14.3	6	
*CqMIC-Dt3-2*	*WqMIC-Dt3-2*	6.76	10.04	0.23	17.4	16.8–18.9	6	
	*IqMIC-Dt3-2*	5.53	7.69	0.21	17.0	16.5–18.5	7	
*CqMIC-Dt8-1*	*IqMIC-Dt8-1*	4.65	6.54	–0.16	16.5	13.5–17.5	6	Guo et al., 2007; Yu et al., 2011; Li C. et al., 2016; Fang et al., 2017; Jia et al., 2018; Liu et al., 2018; Tan et al., 2018
	*WqMIC-Dt8-1*	4.27	6.17	–0.13	16.3	14.4–19.4	4	
*CqMIC-Dt8-2*	*WqMIC-Dt8-2*	3.88	5.59	–0.11	22.4	20.5–26.7	1	Guo et al., 2007
*CqMIC-Dt10-1*	*IqMIC-Dt10-1*	3.84	6.17	0.16	69.5	68.5–70.5	2	
	*WqMIC-Dt10-1*	4.39	7.20	0.19	70.0	68.4–71.5	2	
*CqMIC-Dt10-2*	*WqMIC-Dt10-2*	5.19	8.61	0.20	80.8	80.1–81.5	1	
*CqMIC-Dt10-3*	*WqMIC-Dt10-3*	4.01	6.75	0.19	88.5	88.1–88.8	1	
*CqMIC-Dt11-1*	*WqMIC-Dt11-1*	4.43	7.06	0.26	22.9	22.3–23	1	
*CqMIC-Dt11-2*	*WqMIC-Dt11-2*	4.04	6.60	0.26	32.4	30.4–33.9	1	
*CqMIC-Dt11-3*	*WqMIC-Dt11-3*	4.21	6.12	–0.20	55.7	55–55.7	1	
	*IqMIC-Dt11-1*	3.59	5.41	–0.19	55.0	54.5–55.5	1	
*CqMIC-Dt12-1*	*WqMIC-Dt12-1*	5.83	9.35	–0.22	71.2	65.6–72.9	2	Hulse-Kemp et al., 2015; Huang et al., 2017; Jia et al., 2018; Latyr et al., 2018
*CqMIC-Dt12-2*	*WqMIC-Dt12-2*	3.71	7.90	–0.22	80.5	76.3–86.9	1	

*^a^Iq and Wq refer to QTLs from the methods ICIM-ADD and CIM, respectively; Cq refers to a consensus QTL identified by both methods; Add and PVE represent the additive effect and explanation of phenotypic variation, respectively.*

### Meta-Quantitative Trait Locus Analysis of Micronaire Quantitative Trait Loci

Ten of the 21 MIC QTLs by CIM shared overlapping confidence intervals with those reported in previous studies, including four QTLs that shared overlapping confidence intervals with those in at least three reported studies (Supplementary Table 2). Seven of 12 QTLs identified via ICIM shared overlapping confidence intervals with those reported previously, four of which shared overlapping confidence intervals with those in at least three studies (Supplementary Table 3). Taking together, of the 25 MIC QTLs detected in this study, 13 were new and 12 were previously reported. The results indicate both the reliability and novelty of the current study.

Because the two commonly detected stable QTL (*qMIC-D03-2* on D03 and *qMIC-D08-1* on D08) were also reported in previously studies, their chromosomal regions were identified at 34758451–36484185 bp for *qMIC-D03-2* and at 57060908–61064240 bp for *qMIC-D08-1* based on Zhang et al. (2015). However, to better understand the genes in the two regions, *qMIC-D03-2* were mapped at 42175014–43988973 bp and *qMIC-D08-1* at 60300565–63949530 bp on the two chromosomes based on the updated TM-1 genome sequence (Hu et al., 2019), which were used for the subsequent analysis.

### Gene Ontology Enrichment Analysis of *qMIC-D03-2* and *qMIC-D08-1*

**FIGURE 3:**
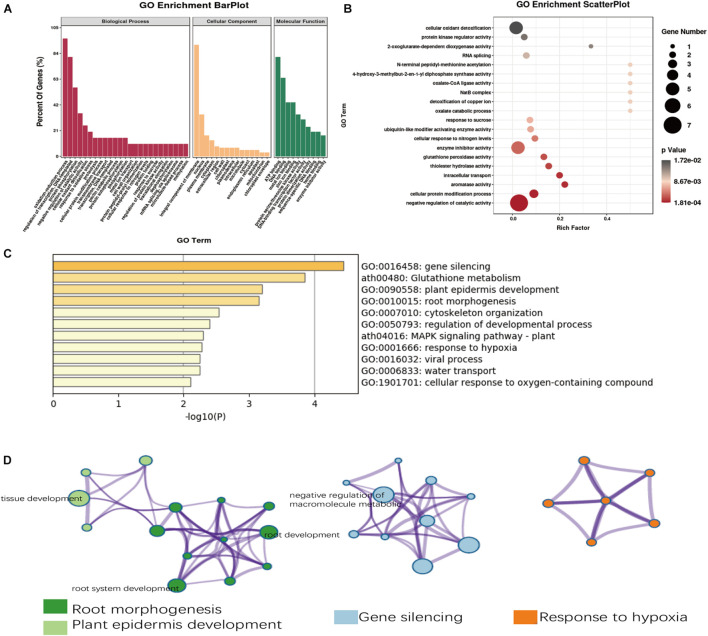
Gene Ontology (GO) analysis of candidate genes of fiber micronaire. **(A)** The annotation of the candidate genes in the two QTLs through GO analysis. **(B)** Top 20 GO terms enrichment in the molecular function category. **(C)** Enriched ontology clusters of fiber micronaire by Metascope. **(D)** The network layout of four significant clusters by Metascope.

Within the chromosomal regions of the two MIC QTLs (*qMIC-D03-2* on D03 and *qMIC-D08-1* of D08), there were 338 predicted genes, and 218 of them had GO annotations (Supplementary Table 4). Based on the GO analysis on the 218 genes, 161 genes were associated with the biological process category, 34 genes were associated with the cellular component category, and 23 genes were associated with the molecular function category. In these three categories, the oxidation-reduction process, integral component of membrane and ATP binding were the most enriched subcategories (Figure 3A). Remarkably, negative regulation of catalytic activity was the most significantly enriched process according to the GO functional enrichment analysis (Figure 3B). For 306 of the 338 putative genes with homologous in *Arabidopsis*, gene silencing, glutathione metabolism, plant epidermis development and root morphogenesis were found to be the main ontology clusters (Figure 3C). These four significant clusters were selected and converted into three network layouts (Figure 3D). It was found that root morphogenesis and plant epidermis development cluster identities were linked. Tissue development, root system development and root development were the main terms and were more proportional to the 306 genes. Negative regulation of macromolecule metabolism and negative regulation of both gene expression and of metabolic process terms were the main terms associated with the gene silencing clusters.

### Prediction of Candidate Genes Within *qMIC-D03-2* and *qMIC-D08-1*

**FIGURE 4:**
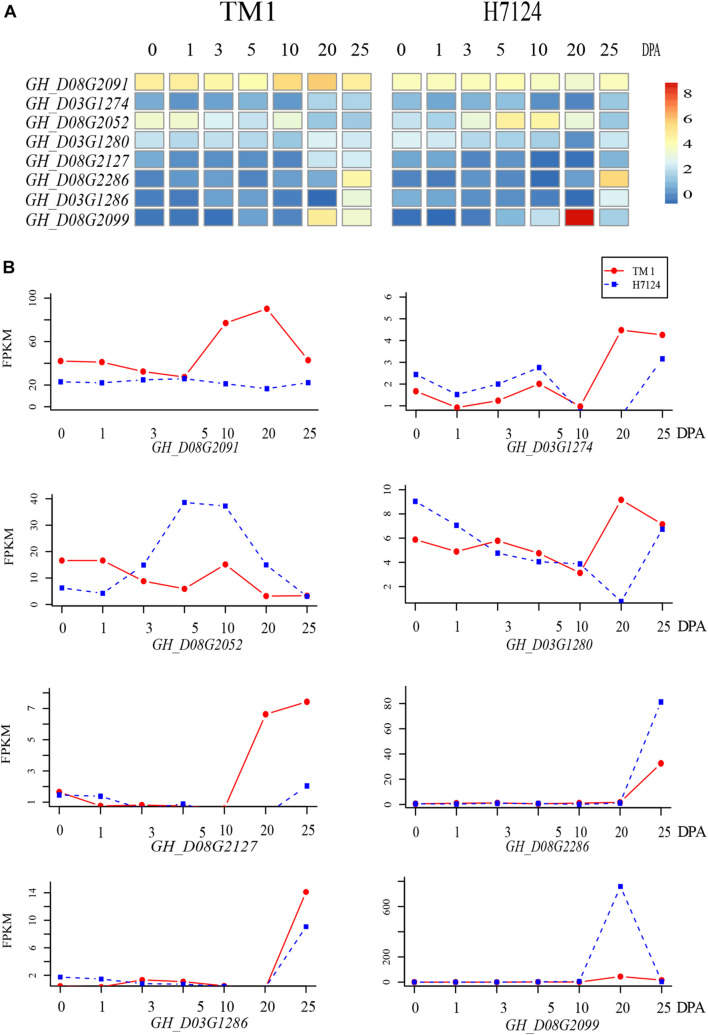
Heat map and trend plot of the expression of candidate genes detected within two stable QTLs related to MIC. **(A)** Heatmap of the RNA-seq data (log_2_(1 + RPKM)) of eight candidate genes during fiber development. **(B)** Line charts of genes listed in the left heatmap from top to bottom.

Because both the *G. hirsutum* TM-1 and *G. barbadense* Hai7124 genomes were sequenced and CRI35 is a typical upland cotton cultivar, The expression levels of the 338 genes from TM-1 and Hai7124 in the two QTL regions were determined based on existing RNA sequencing (RNA-seq) data (the National Genomics Data Center: https://bigd.big.ac.cn/bioproject/; accession number: PRJNA490626) (Hu et al., 2019). The fold change in candidate gene expression was set to 2 as the threshold for significant differential expression between TM-1 and Hai7124 in corresponding tissues including embryos (0, 1, 3, and 5 DPA) and fibers (10, 20, and 25 DPA). As a result, eight candidate genes (three genes for *qMIC-D03-2* and five genes for *qMIC-D08-1*) were found to be differentially expressed between TM-1 and Hai7124 (Figure 4). The following is a detailed *in silico* analysis of those eight genes.

For the three candidate genes on chromosome D03, *GH_D03G1280* and *GH_D03G1274* encode a kinase superfamily protein and the NADPH/respiratory burst oxidase protein D, and the expression levels of both genes in TM-1 were higher than that in Hai7124 at 20 DPA. *GH_D03G1280* had SNP variants including frameshift variants and synonymous variants between TM-1 and Hai7124. Calcium-dependent NADPH oxidase generates superoxide molecules, a reactive oxygen species (ROS). The third gene, *GH_D03G1286* encodes a transducin/WD40 repeat-like superfamily protein and its expression in both TM-1 and Hai7124 during the early stages of fiber development was low until 20 or 25 DPA. This gene had SNP variants between TM-1 and Hai7124 including loss/gain of a stop codon and splice region variants and GAA frameshift variants which might be involved in the change in fiber secondary cell wall synthesis.

*GH_D08G2052* encodes a TCP family transcription factor, and its expression was significantly higher in Hai7124 than in TM-1 during fiber elongation from 5 to 20 DPA. Three SNP variants (frameshift variant, loss of a stop codon, and splice region types) were found between TM-1 and Hai7124, which might be involved in the change in fiber elongation. *GH_D08G2091* encodes a glutathione S-transferase THETA 1 enzyme and is a homolog of *AT5G41210* in *Arabidopsis*, and its expression was significantly higher in TM-1 than in Hai7124 during fiber development from 10 to 25 DPA, when fast elongation and secondary cell wall synthesis occurred. *GH_D08G2127* encodes a receptor-like kinase in flowers and is a homolog of *AT2G48010* in *Arabidopsis*, and the expression level in TM-1 was higher than that in Hai7124 at 20 and 25 DPA. Similar to *GH_D03G1274*, the protein encoded by the gene is involved in protein phosphorylation.

*GH_D08G2099* and *GH_D08G2286* encode beta-6 tubulin protein and xyloglucan endo-transglycosylase-related 8, respectively. The expression of these two genes was very low in the early stage of fiber development in both TM-1 and Hai7124 until 20 or 25 DPA. The expression levels of *GH_D08G2099* (17 times higher) and *GH_D08G2286* in Hai7124 fibers were higher than those in TM-1 fibers at 20 and 25 DPA, respectively. The upstream region of *GH_D08G2286* lacked a 3126 bp fragment at −5393 bp in Hai7124 but not in TM-1. A detailed description of sequence variation for all the eight genes is listed in Supplementary Table 5.

Based on predictions of miRNA target genes by psRNATarget, *GH_D08G2286* was the target gene of *ghr-miR156a*, *ghr-miR156b*, and *ghr-miR156d*, and the average expression level of these three miRNAs was 289.11 FPKM. *GH_D03G1286* was the target gene of *ghr-miR164*, and the expression level of *ghr-miR164* was 2114 FPKM.

### Identification of Co-segregating Markers for Micronaire

Stable MIC QTLs are important loci shaping MIC, and the closed linked markers are valuable for MAS. For the two stable MIC QTLs, *qMIC-D03-2* and *qMIC-D08-1*, Markers 150834 and 175863 were the nearest SNPs, respectively (Supplementary Figure 2A). For marker 150834 for *qMIC-D03-2*, the BILs with the SNP allele genotype (AA) from CRI36 averaged a significantly greater MIC value than did those with the SNP allele genotype (TT) from Hai7124 (4.30 vs. 3.89, *P* < 0.05). However, for marker 175863 for *qMIC-D08-1*, the BILs with the SNP allele genotype (AA) from CRI36 averaged a significantly lower MIC value than did those with the SNP allele genotype (GG) from Hai7124 (3.95 vs. 4.25, *P* < 0.05). The QTL allele for *qMIC-D03-2* had a greater additive effect (−0.21) than that from *qMIC-D08-1* (−0.15), consistent with the early QTL analysis (Table 2). MIC for the desirable QTL genotype for D03 without the desirable genotype for D08 (i.e., *Q_3_Q_3_q_8_q_8_*) was 4.06, vs. 4.16 for the desirable QTL genotype for D08 without the desirable genotype for D03 (i.e., *q_3_q_3_Q_8_Q_8_*). When the desirable alleles from the two QTLs were combined into the same genotype (*Q_3_Q_3_Q_8_Q_8_*, i.e., TT for *qMIC-D03-2* with AA for *qMIC-D08-1*), MIC was further reduced to 3.73 (significantly lower than that from *q_3_q_3_Q_8_Q_8_* but not from *Q_3_Q_3_q_8_q_8_*), as compared to 4.44 for the genotype without any desirable allele (i.e., *q_3_q_3_q_8_q_8_*). The effects from the two QTLs were additive and there appeared no interaction between them (Supplementary Figure 2D). Furthermore, the two homozygous genotypes for each of the two SNP markers (150834 and 175863) had similar fiber length and strength (Supplementary Figures 2B,C), indicating that these two QTLs did not affect fiber length and strength. Therefore, these two SNPs could be used to design portable markers for MAS to improve MIC without affecting fiber length and strength.

## Discussion

Micronaire is measured as the air permeability of a compressed lint sample of known mass and is essentially the fiber weight per unit length (μg inch^–1^) for a single fiber. Therefore, lint yield improvement through breeding has been accompanied by the increase of MIC (Zhang et al., 2019). Therefore, it is not surprising that the MIC of new cultivars has been increased, because of the positive correlation between lint yield and MIC. Sun et al. (2019) showed that a global collection of 719 upland cotton germplasm accessions only had very low percentage of lines with the premium MIC (i.e., 3.70–4.20). As lint with MIC higher than 5.0 will suffer price discounts, breeding for low fiber MIC is becoming increasingly important.

### Populations From Parents With Low Fiber Micronaire Could Be Used for Quantitative Trait Locus Analysis

In the nine testing environments, Hai7124 and CRI36 had MIC ranges of 3.12–4.22 and 3.27–4.50, respectively. Both parents had low MIC and were considered degree A MIC in China according to the national standards. The results indicated that both upland cotton CRI36 and Egyptian cotton Hai7124 possessed genomic regions that could decrease MIC. However, the upland parent still had significantly higher MIC. Therefore, it was still valid to carry out the current QTL analysis in the BIL population developed from the two parents. The results further showed that the two parents of different species possessed different genetic loci involved in MIC formation in that both parents had QTL alleles decreasing MIC (8 QTLs in CRI36 vs. 17 QTLs in Hai7124), consistent with many previous QTL studies in cotton (Lacape et al., 2010; Zhang et al., 2014, 2020; Zhu et al., 2020). As such, transgressive segregation in MIC was observed in that the BILs developed from the two parents had MIC ranging from 2.10 to 6.17, with an average BLUP of 4.18. Moreover, a very large proportion of the BIL population (35.6% of the BILs) had a degree A MIC. These lines with QTL introgression for low MIC and the desirable QTL alleles and their linked markers should be useful in MAS for breeding cotton with a premium fiber quality.

### Utilization of Best Linear Unbiased Predictions With Different Principal Component Analysis Clusters and the Complementation of the Two Quantitative Trait Loci Mapping Methods

In this study, results showed that different ecological environments had a great influence on MIC. The MIC of the BILs grown in the Northwestern Inland Valley averaged 0.5 lower than these grown in the Yellow River Valley. Hence, the BLUPs of the PCA cluster, which represented the two different ecological environments, could be used to identify QTLs associated with specific ecological environments. These QTLs may be useful in MAS of low MIC for a particular ecological environment. Interestingly, QTL *CqMIC-At11-1* was identified in blup region one test and in six individual tests. This indicated that it was necessary to analyze QTLs with BLUPs associated with different ecological environments and that the results were reliable. Therefore, when there is a genotype × environment interaction for a trait of interest in a multi-location experiment, testing environments can be grouped for a BLUP analysis for each group instead of using overall means in QTL mapping, in addition to a separate analysis for each environment. In this study, grouping based on PCA did not completely reflect geographical regions of tests because one test in Anyang, Henan, 2015, of Yellow River Valley and another test in Sanya, Hainan, 2016 were grouped with the Northwestern Inland Valley (i.e., Xinjiang). In addition to soil type, soil fertility and moisture (Hearn, 1994), and crop management practices, it is known that MIC is greatly influenced by weather conditions including daily temperature (especially night temperature) and relative humidity (Gipson and Joham, 1968; Wanjura and Barker, 1985; Liakatas et al., 1998; Reddy et al., 1999; Bange et al., 2010). We speculate that the dry periods with low temperatures during the boll development stage in the two tests were likely the major cause for decreased MIC, as frequently observed in Xinjiang.

In this study, 13 and 4 specific QTLs were identified by CIM and ICIM, respectively. However, eight common QTLs were identified via both QTL mapping software programs. Both methods can identify common chromosomes with QTLs, and most of the QTLs (67.7%) of the MIC QTLs detected by ICIM were also detected by CIM, while the remaining unique QTLs detected by ICIM differed in mapping positions from these detected by CIM on the same chromosomes. CIM can detect more QTLs on the same chromosomes and may be more QTLs on additional chromosomes. Therefore, CIM is more powerful in detecting QTL, as proposed by Zeng (1993, 1994) when the CIM method was developed. The results demonstrate that both mapping methods are useful and are complementary to one another to detect additional QTL loci. Common QTLs detected by the two methods provide some levels of confidence in mapping results. Therefore, we suggest that the two QTL mapping methods be simultaneously used. Of course, common QTLs especially these with major effects should be focused in further studies.

Another important aspect is if some of the MIC QTLs detected in this study were also overlapped with QTLs for lint yield and fiber length and strength, leading to MIC’s correlation with lint yield, fiber length and strength. Overlapped QTL regions for these traits are likely due to linked genes or pleiotropic effects of genes for the traits, which would explain the correlations of MIC with the three traits. A subsequent QTL analysis will be performed to address these questions.

### Gene Ontology Enrichment and Candidate Gene Identification

In this study, two methods were used to perform GO analysis of putative genes with the two common QTL regions (*qMIC-D03-2* and *qMIC-D08-1*). The results showed that the oxidation-reduction process, integral component of membranes and ATP binding were the most populated subcategories. Root morphogenesis, plant epidermis development, gene silencing and response to hypoxia were the main clusters according to Metascape. The results of the two methods coincided and showed that the following hypothesis governing MIC by the two QTLs: During fiber elongation, fiber cells are hypoxic, giving rise to a response to hypoxia that negatively regulates enzymatic catalytic activities to induce fiber morphogenesis. This was followed by secondary cell wall synthesis and changes in membrane components, eventually leading to a change in MIC. This hypothesis was supported by the finding that immature fiber mutants had reduced ROS levels and reduced energy production in developing fibers compared with mature fibers (Kim et al., 2013).

In this study, eight candidate genes were identified for the two QTL regions. Xyloglucan might negatively affect fiber elongation according to comparisons of xyloglucan contents between *G. barbadense* and *G. hirsutum* (Li et al., 2013). *GH_D08G2286* encodes xyloglucan endo-transglycosylase-related 8 (GhXTR8) and has a function similar to that xyloglucan endo-transglycosylase/hydrolase (XTH) proteins, which, when overexpressed in cotton plants, result in 15–20% longer fiber compared with that of wild-type cotton (Lee et al., 2010). *GH_D03G1298* encodes a glucuronoxylan 4-O-methyltransferase-like protein (DUF579) that is involved in xyloglucan metabolism and that is located within the *qMIC-D03-2* region. The expression of the genes encoding both of these proteins in Hai7124 was higher than that in TM-1 at 25 DPA. *DUF579* was also determined to be involved in xylan biosynthesis according to phylogenetic analysis (Chen et al., 2020). *IRX15* and *IRX15-L* are homologous genes of *DUF579* in *Arabidopsis*; and characterization of a double knockout line revealed irregular secondary cell wall margins of fiber cells and a lower degree of xylan polymerization compared with that of the wild-type line (Jensen et al., 2011).

*GH_D03G1280* (a protein kinase superfamily gene) was also reported to participate in fiber elongation (Li C. et al., 2016). The protein coded by *GH_D03G1286* belongs to a WD40 protein superfamily and mainly regulates the formation of trichomes via the R3 *MYB-bHLH-WD40* transcriptional complex in Arabidopsis (Gan et al., 2011), but a divergent WD40 protein (*GhWDR*) interacts with GhMML4_D12 in a process similar to but different from that of the MBW transcriptional complex involved in trichome development (Tian et al., 2020). The protein coded by *GH_D08G2052* is a *TCP* family transcription factor, and *GhTCP4* plays an important role in balancing cotton fiber elongation and cell wall synthesis together with miR319 (Cao et al., 2020). *GH_D08G2099* encodes a beta-6 tubulin protein that is involved in fiber development. Nineteen beta-tubulin cDNAs were detected in developing cotton ovules and were found to be highly expressed in elongating fiber cells (He et al., 2008). Beta-tubulin was also identified by QTL analysis and was found to control fiber quality (Guo et al., 2021). *GH_D03G1274* encodes NADPH/respiratory burst oxidase protein D (*RBOHC*), and *AtRBOHC* influences the development of root hairs via the activation of Ca^2+^ and K^+^ osmotic pathways in plant root cells (Bai et al., 2014). RBOHC may mediate the progression of ABA-regulated primary root growth by producing ROS in the roots (Ma X. et al., 2019). *GH_D08G2091*, which encodes a glutathione transferase, also regulates the production of ROS. The products of both *GH_D03G1274* and *GH_D08G2091* participate in ROS metabolic pathways, and ROS can act as developmental signaling molecules in the process of secondary cell wall differentiation in cotton fibers (Li et al., 2007; Guo et al., 2016). *GH_D03G1262* encodes an ARF-GAP domain 1 protein (*AGD1*), which regulates root hair polarity by coordinating cytoskeleton and membrane trafficking (Yoo and Blancaflor, 2013). To determine which of these 8 genes contribute to MIC within the two QTL regions, further studies are needed.

It is recognized that only one specific candidate gene in each of the two MIC QTL regions will be the one determining a proportion of the genetic differences in MIC between the two parents. Functions of other genes within the two QTL regions are most unlikely associated with MIC and should not be overstated. Although other molecular aspects including quantitative RT-PCR between parents and BILs with contrasting MIC and virus-induced gene silencing can be performed for those 8 genes, further high resolution mapping using a larger interspecific genetic population is required. In addition, the desirable effect (reducing MIC) for *qMIC-D03-2* was from the allele contributed from the Egyptian Hai7124 cotton. Therefore, the two QTLs may be specific to interspecific hybrid populations between the two species. A panel of upland cotton germplasm lines would not be useful in validating the QTL effect. Near-isogenic lines will be developed for the two QTL regions for a more in-depth analysis in the future.

In summary, an interspecific BIL population of 250 lines from *G. hirsutum* × *G. barbadense* was employed to detect MIC QTLs in nine replicated field tests. Based on a high-density genetic map with 7709 genotyping-by-sequencing (GBS)-based SNP markers, 25 MIC QTLs were identified, including 12 previously described QTLs and 13 new QTLs. Importantly, eight candidate genes within two stable MIC QTL regions were identified with differential expression between upland TM-1 and Egyptian Hai7124. This study provides valuable information for improving MIC in cotton breeding.

## Data Availability Statement

The datasets presented in this study can be found in online repositories. The names of the repository/repositories and accession number(s) can be found in the article/Supplementary Material.

## Author Contributions

WP analyzed and summed all the data and wrote the manuscript. JS performed the gene expression and wrote introduction of the manuscript. WW, MW, QC, QQ, CH, HL, XG, HH, and YZ managed and collected the phenotype data. JM, BJ, and LW involved in the analysis of SNP markers. YQ and JY directed the experiments. JZ revised the manuscript. All authors read and approved the final manuscript.

## Conflict of Interest

XG, HH, and YZ are employed by Join Hope Seed Co., Ltd., Changji, China. The remaining authors declare that the research was conducted in the absence of any commercial or financial relationships that could be construed as a potential conflict of interest.

## Publisher’s Note

All claims expressed in this article are solely those of the authors and do not necessarily represent those of their affiliated organizations, or those of the publisher, the editors and the reviewers. Any product that may be evaluated in this article, or claim that may be made by its manufacturer, is not guaranteed or endorsed by the publisher.

## Footnotes

1
www.cottonqtldb.org

2
https://www.omicstudio.cn/tool

3
http://plantgrn.noble.org/

4
http://cotton.zju.edu.cn/

## Supplementary Material

Supplementary Figure 1 Frequency distribution of fiber micronaire of 250 BILs in different environments. 15Aync, 16Aync, and 17Aync represent the environment of Anyang south farm in 2015, 2016, and 2017, respectively; 16Xjal and 17Xjal represent the environment of Alaer, Xinjiang in 2016 and 2017, respectively; 17Aydc,17Hbwx, and 17Xjsh represent the environment of east farm, Anyang, Henan, Weixian, Hebei, and Shihezi, Xinjiang in 2017; 16Hnsy represent the environment of Sanya, Hainan in 2016.

Supplementary Figure 2 The QTL allele effect for the *qMIC-D03-2* and *qMIC-D08-1* related to MIC, FL, and FS. **(A)** Box plots for the *qMIC-D03-2* and *qMIC-D08-1* related to MIC. **(B)** Box plots for the *qMIC-D03-2* and *qMIC-D08-1* related to FL. **(C)** Box plots for the *qMIC-D03-2* and *qMIC-D08-1* related to FS. **(D)** The QTL allele effect for *qMIC-D03-2* and *qMIC-D08-1* related to MIC.
