# Supplementary figures and images for: Quantitative Trait Locus Analysis and Identification of Candidate Genes for Micronaire in an Interspecific Backcross Inbred Line Population of Gossypium hirsutum × Gossypium barbadense
Source: Front Plant Sci. 2021 Oct 27;12:763016. doi: 10.3389/fpls.2021.763016 (PMC8579039; doi:10.3389/fpls.2021.763016)

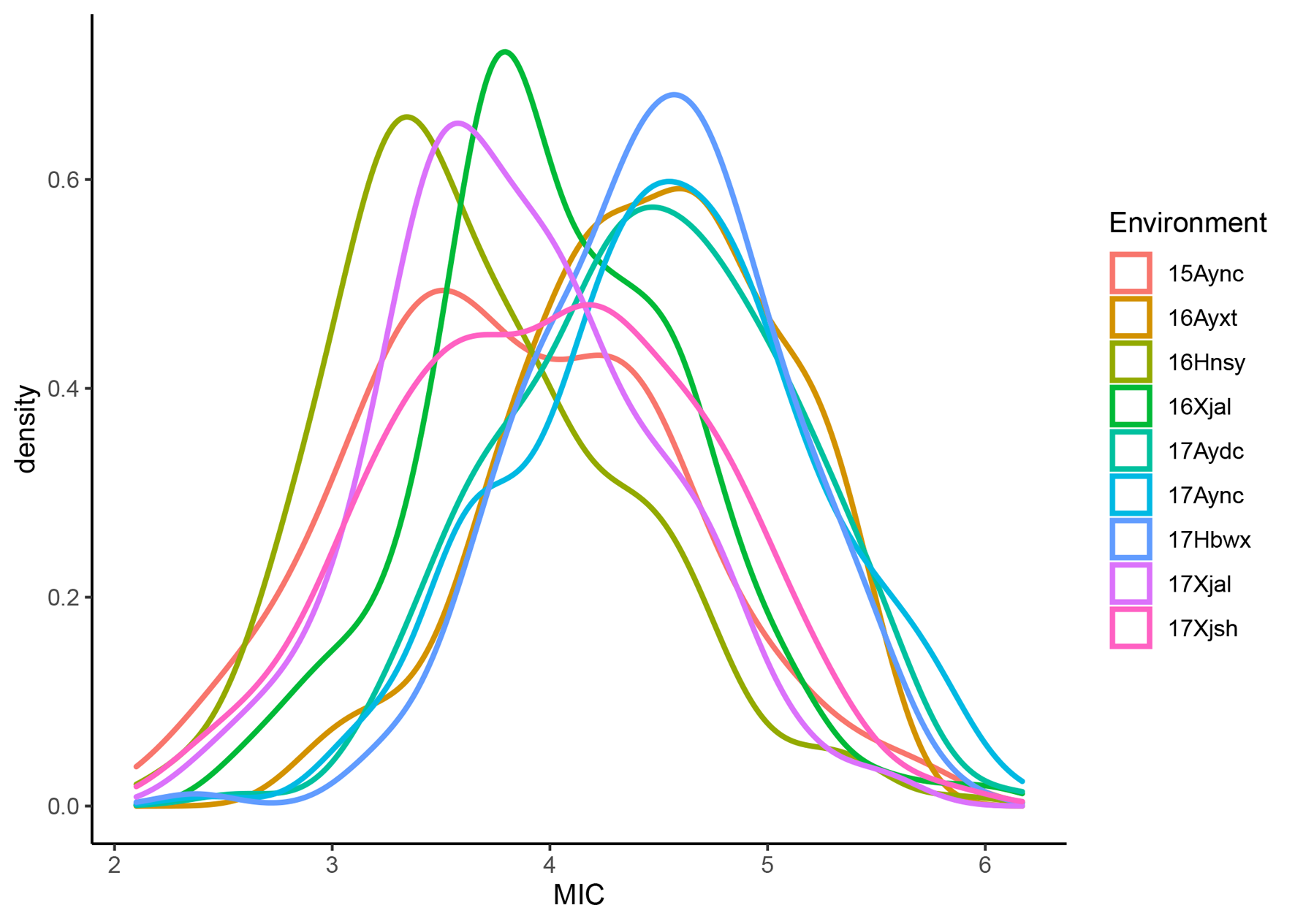

Supplement: Supplementary Figure 1 — Frequency distribution of fiber micronaire of 250 BILs in different environments. 15Aync, 16Aync, and 17Aync represent the environment of Anyang south farm in 2015, 2016, and 2017, respectively; 16Xjal and 17Xjal represent the environment of Alaer, Xinjiang in 2016 and 2017, respectively; 17Aydc,17Hbwx, and 17Xjsh represent the environment of east farm, Anyang, Henan, Weixian, Hebei, and Shihezi, Xinjiang in 2017; 16Hnsy represent the environment of Sanya, Hainan in 2016. [file Image_1.TIF]

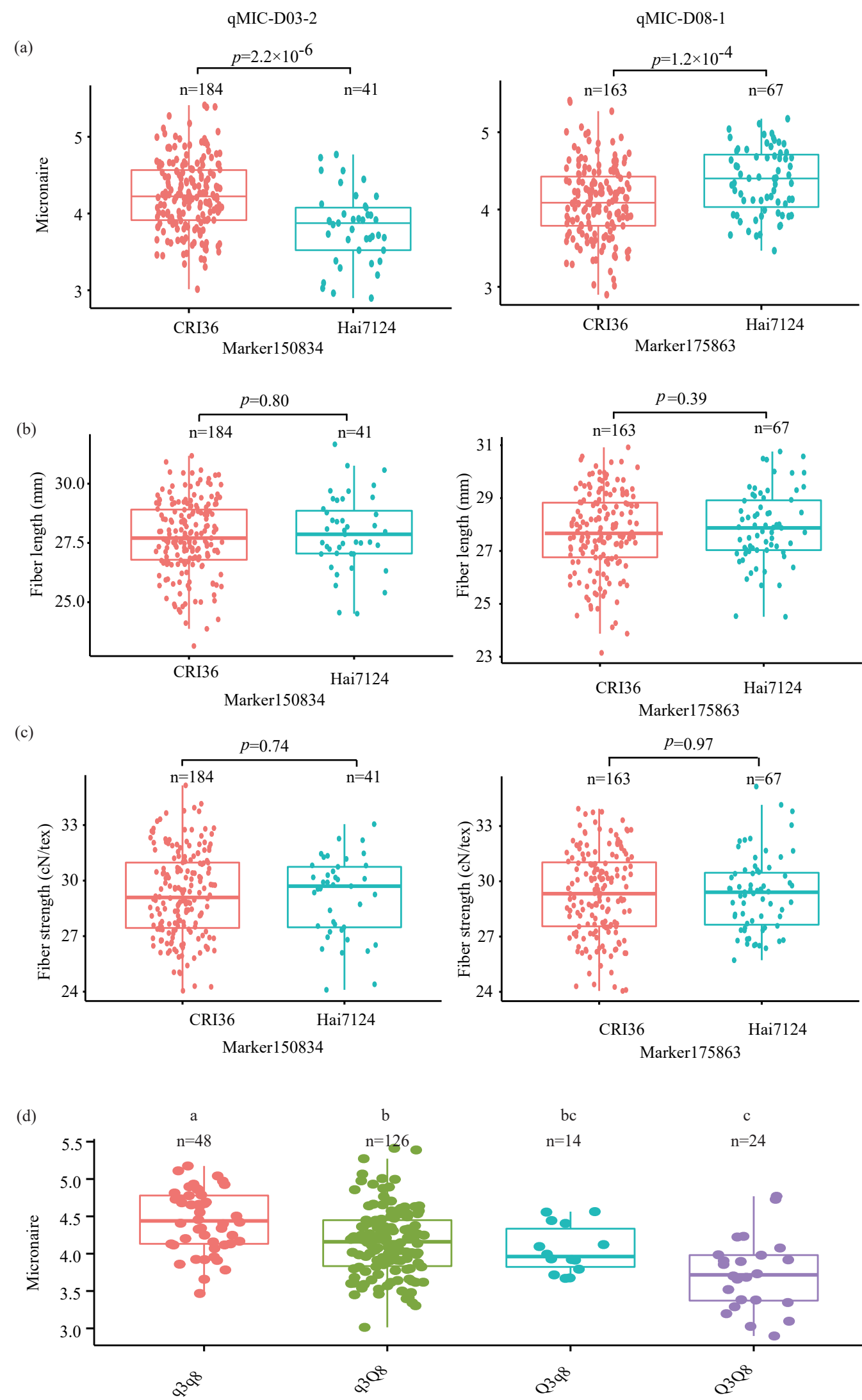

Supplement: Supplementary Figure 2 — The QTL allele effect for the qMIC-D03-2 and qMIC-D08-1 related to MIC, FL, and FS. (A) Box plots for the qMIC-D03-2 and qMIC-D08-1 related to MIC. (B) Box plots for the qMIC-D03-2 and qMIC-D08-1 related to FL. (C) Box plots for the qMIC-D03-2 and qMIC-D08-1 related to FS. (D) The QTL allele effect for qMIC-D03-2 and qMIC-D08-1 related to MIC. [file Image_2.pdf]
